# Acetyl-glucomannan from *Dendrobium officinale*: Structural modification and immunomodulatory activities

**DOI:** 10.3389/fnut.2022.1016961

**Published:** 2022-09-29

**Authors:** Xiaoyu Guo, Mingguan Yang, Changlu Wang, Shaoping Nie, Steve W. Cui, Qingbin Guo

**Affiliations:** ^1^State Key Laboratory of Food Nutrition and Safety, College of Food Science and Technology, Tianjin University of Science and Technology, Tianjin, China; ^2^College of Food Science and Engineering, Qilu University of Technology (Shandong Academy of Sciences), Jinan, China; ^3^State Key Laboratory of Food Science and Technology, China-Canada Joint Laboratory of Food Science and Technology (Nanchang), Nanchang University, Nanchang, China; ^4^Guelph Research and Development Centre, Agriculture and Agri-Food Canada, Guelph, ON, Canada

**Keywords:** *Dendrobium officinale* polysaccharide, O-acetylation, immunomodulatory, structural modification, glucomannan

## Abstract

To understand the mechanisms of immunomodulatory effect, *Dendrobium Officinale* polysaccharides (DOP) were treated by ultrasound and mild base separately to generate fractions of various weight-average molecular weight (Mw) and degrees of acetylation (DA). The structural features, conformational properties, functional properties and immunomodulatory activities of original and modified DOPs were investigated. Ultrasonic treatment decreased the Mw and apparent viscosity and improved the water solubility of DOP. Mild base treatment remarkably reduced the DA and the water solubility, while the overall apparent viscosity was increased. Conformational analysis by triple-detector high performance size-exclusion chromatography showed that the molecular chain of DOP turned more compact coil conformation with decreased DA. Results from the macrophages RAW 264.7 analysis showed that samples sonicated for 200 min (Mw 34.2 kDa) showed the highest immune-regulation effects. However, the immunomodulatory effects of the samples after de-acetylation were all compromised compared to the original DOP. This study inspires further research to establish the structural-immunomodulatory relationships, which promote the application of DOP in both the food and medicine fields.

## Introduction

*Dendrobium officinale* polysaccharides (DOP) have been successfully discovered and widely used in healthy food and medicine due to the broad spectrum of their biological properties. Many *in vitro* and *in vivo* studies indicated that DOP had immunomodulatory, antitumor, maintenance of colonic health, gastro-protective, hypoglycemic, antiinflammatory, antioxidative, antimutagenic, hepatoprotective, and vasodilating effects (1–3). These effects are closely related to various structural characteristics of DOP, e.g., molecular weight, higher-order structure (i.e., conformation), functional groups, and degree of branching (4, 5). DOP is a linear glucomannan consisting of D-mannose and D-glucose linked by β-1,4 glycosidic bonds at varied ratios. Acetyl groups in DOP have been identified to attach to O-2 and O-3 of mannan in the majority of mono-substituted and small amounts of di-substituted forms (6, 7). The M_*W*_ of DOP has been reported to be varied from 8.5 to 399 kDa (4).

A variety of biological activities, such as immunomodulating and immuno-pharmacological activities, are observed with DOP. DOP plays a vital role in regulating the immune system by strengthening one or several nonspecific immune responses, cellular-mediated immune responses, and humoral immune responses (4). Results of recent *in vitro* experiments on different kinds of murine or human cells (dendritic cells, spleen cells, macrophage cells, and THP-1 cells) demonstrated that DOP could promote cell viability, NO production, and cytokine secretion (TNF-α, IL-1β). Some *in vivo* studies have shown that DOP could stimulate the proliferation of splenocytes, balance the ratio of spleen lymphocyte subsets and the secretion of serum cytokines, up-regulate the serum IgM, IgG, and haemolysin formation, and accelerate the phagocytotic function of peritoneal macrophage (8–10). The immune response exerted by DOP was reported to be mediated through the TLR-4 signaling pathway (8). However, detailed information regarding the relationship between the molecular structure of DOP and its immunomodulatory effects remains scant.

To bridge the gap between the molecular structure and immunomodulatory effects of DOP, structural modification is not neglectable. Different molecular degradation and derivatization methods have been used, including alkylation, carboxymethylation, sulfation, selenylation, phosphorylation, ultrasonic disruption, and the degradation of polysaccharides, which are generally classified as chemical, physical, and biological modifications (11). However, finding a suitable modification method is difficult. For example, decreasing the Mw of polysaccharides without affecting the other structural features, e.g., degree of branching or monosaccharide composition, is challenging. Recently, our team have tried trifluoroacetic acid (TFA) hydrolysis, xylanase treatment, and ultrasonication treatment to degrade arabinoxylan from wheat bran. Results indicated that TFA favored the removal of the arabinose side chain; xylanase treatment results in two fractions with different Mw; only ultrasonication treatment could decrease the Mw without affecting the overall degree of branching and solubility of arabinoxylan (12). Similarly, to understand the effects of DA on the bioactive properties of DOP, either acetylation or de-acetylation needs to be conducted to obtain samples of varied DA. However, the acetylation reaction can not control the reaction sites, given that the acetyl groups of naturally occurring DOP locate only on O-2 and/or O-3 positions of mannose residues (7). For de-acetylation reaction, strong bases such as NaOH solution excludes most acetyl groups altogether, even under a small concentration and short time duration. The produced DOPs by NaOH treatment contains only trace amount of acetyl groups, leading to poor water solubility (13). Therefore, mild bases, e.g., Na_2_CO_3_, were gradually used to remove the acetyl group to get DOP fractions with varied DA values (14).

In this study, DOP was degraded and de-acetylated using the ultrasonic and Na_2_CO_3_ treatments separately. The structural features, conformational properties, functional properties, and immunomodulatory activities of native and modified DOPs were investigated, aiming to understand their structure-bioactivity relationships and facilitate applications in health and functional food areas.

## Materials and methods

### Materials

The RAW 264.7 macrophages were obtained from the key laboratory of food nutrition and safety, Tianjin University of Science and Technology, Tianjin, China. The monosaccharide standards (D-Glucose, D-Xylose, L-Rhamnose, D-Mannose, L-Arabinose, and D-Galactose) were purchased from Sigma Chemical Co. (St. Louis, MO, USA). The ELISA kits were purchased from Multisciences (Lianke) Biotech Co. Ltd. (Hangzhou, China). All other chemical reagents and solvents purchased were all analytical grade unless otherwise stated.

### Extraction and purification of *Dendrobium Officinale* polysaccharides

The extraction and purification of DOP were conducted according to Wang et al. with slight modifications (15). Briefly, dry powder of the *D. officinale* herbal (250 g) was suspended in 95% ethanol (250 ml) in a beaker with constant stirring for 24 h. The suspension was subjected to two more cycles of 24-h soaking and subsequent centrifugation at 10,000 *g* and 25°C for 20 min. Then, the residue was extracted with water (1:20 w/v) stirred at 70°C for 4 h followed by centrifugation at 10,000 *g* and 25°C for 20 min. The obtained supernatants were concentrated (to 1/4 of the original volume) using a rotary evaporator, and then ethanol precipitated (1:3 ratio, v/v) at room temperature to accumulate the crude polysaccharide. Subsequently, thermostable α-amylase (3,000 units/ml) was added to 1% polysaccharide solution, stirred at 70°C for 2 h, and then cooled to room temperature. The small molecular weight contaminants produced by the hydrolysis were removed by dialysis against deionized water (with 3,500 Da cut-off) for 72 h. The solution was further freeze-dried to obtain a dry sample of purified polysaccharide (DOP).

### Chemical composition analysis

The total sugar content was determined by the phenol-sulfuric acid method with glucose as the standard (16). The soluble protein was determined using the Ninhydrin colorimetry method (Amino acid detection kit, Solarbio) (15).

### Molecular modification of *Dendrobium Officinale* polysaccharides

The ultrasonic treatment of DOP was performed according to Striegel et al. with slight modifications (17). About 30 ml DOP solution (10 mg/ml) was prepared at room temperature under constant stirring. Ultrasonic treatment was then performed by an ultrasonic homogenizer (JY92-IIN, Ningbo Scientz Biotechnology Corporation, China) under controlled conditions (500 W, 40°C, on for 2 s, off for 1 s) for 60, 200, and 720 min, respectively. The samples were then ethanol precipitated (1:3 ratio, v/v) to obtain the modified DOPs, which were termed as US-60, US-200, and US-720, respectively.

De-acetylation of DOP was carried out according to Tamaki with slight modifications (18). Briefly, DOP (50 mg) was completely dissolved in distilled water (20 ml). After adding an equal volume of 0.2 M Na_2_CO_3_ solution, the suspension was then reacted at 25°C for 3, 5, and 25 min separately with continuous mixing. The mixture was quickly adjusted to pH 4.5 with 1 M HCl, dialyzed against distilled water, and then freeze-dried. These samples obtained were depicted as DA-3, DA-5, and DA-25, respectively.

### Structural characterization

#### Degree of acetylation

The degree of acetylation (DA)of modified DOPs was determined by the titration method according to Huang et al. with slight modifications (19). Twenty milligram of grounded sample was added to the aqueous solution of sodium hydroxide (0.01 M, 10 ml) and kept in the water bath (50°C) for 2 h. The excess alkali was titrated with 0.01 M hydrochloride acid using phenolaphtalen as the indicator. The degree of de-acetylation (DD) was calculated according to the equation as follows (20).


(1) $$DA(\%)=[(V_{0}C_{0}-V_{1}C_{1})\times 0.043\times 100]/M$$

Where V_0_ is the volume of NaOH in ml, C_0_ is the concentration of NaOH in mol/L, V_1_ is the volume of HCl in ml, C_1_ is the concentration of HCl in mol/L, M is the weight of the sample (dry) in g.


(2) $$DD(\%)=\frac{A_{1}-A_{0}}{A_{1}}\times 100$$

*A*_1_ is the acetyl group content of DOP in %, *A*_0_ is the acetyl group content of de-acetylated DOP in %.

#### Methylation analysis

The methylation analysis was performed as described previously (21). Firstly, 3 mg of sample was fully dissolved in dimethyl sulfoxide (0.5 ml) and then dried NaOH powder (20 mg) was added to the solution with stirring for 2 h at room temperature. Methyl iodide (0.6 ml) was then added to fully convert all the free hydroxyl groups into methoxy groups and evaporated by a stream of nitrogen. The dried methylated polysaccharides were hydrolyzed by TFA (4 M), reduced using sodium borodeuteride (4 mg) and acetylated with acetic anhydride (50 μl) to produce the partially methylated alditol acetates (PMAA). PMAAs were then analyzed using GC-MS (Agilent 7890, USA) equipped with an HP-5 column programmed from 160 to 210°C at 2°C/min, and then 210 to 240°C at 5°C/min.

#### FT-IR analysis

FT-IR spectra of samples were obtained according to the KBr disk method. Briefly, 1 mg polysaccharide was ground with 150 mg KBr into a fine powder and then pressed into a pellet, which is measured using the FT-IR spectrometer (Nicolet IS50, USA) in the frequency range of 4,000–400 cm^–1^ with 32 scans at a resolution of 4 cm^–1^.

### Water solubility test

The water solubility was evaluated according to Zhu et al. with slight modifications (22). Briefly, samples (0.5%, w/v) were dispersed in distilled water followed by incubation at 25°C for 90 min and mixing for 5 s every 30 min. The mixture was centrifuged (10,000 *g* and 25°C for 5 min) to collect the insoluble sediment, followed by freeze-drying. The solubility was calculated based on the following equation as follows:


(3) $$\text{Solubility}(\%)=\frac{w_{\text{i}}-w_{\text{p}}}{w_{\text{i}}}\times 100$$

Where *w*_*i*_ is the initial weight of the complete sample in g, and *w*_*p*_ is the weight of the dried sediment in g.

### High performance size-exclusion chromatography analysis

The chain conformation of samples was analyzed using an high performance size-exclusion chromatography (HPSEC) equipped with multi-detectors (multi-angle laser light scattering, refractive index, ultraviolet detector, and online viscometer). Samples were eluted at a flow speed of 0.6 ml/min within Shodex TM OHpak SB-803 HQ (8.0 × 300 mm, 6 μm) and SB-805 HQ (8.0 × 300 mm, 13 μm) columns (Showa Denko K.K., Tokyo, Japan) in series. The columns and detectors were maintained at 40°C. Data was analyzed using the ASTRA 7.1.3 software. A refractive index increment (dn/dc) of 0.146 ml/g was used in the calculation.

### Immunomodulatory assays

#### Cell culture

RAW264.7 macrophages were cultured in RPMI-1640 medium (Gibco, USA) supplemented with 10% fetal bovine serum (Gemini, USA) and 1% penicillin-streptomycin (Hyclone, USA) and incubated at 37°C with a 5% CO_2_ humidified atmosphere in a carbon dioxide cell incubator (Thermo, USA). The cells were stimulated with the control group (without polysaccharides), positive control group (LPS: 2 μg/mL) and various concentrations of DOP and modified DOPs (50, 100, 200, 400, and 800 μg/mL) (23).

#### The proliferation and phagocytosis activity assays

Cells were adjusted to a concentration of 5 × 10^4^ cells/ml, loaded onto the 96-well plates (100 μl/well), and continuously incubated for 12 h. Then, the cells were stimulated with the blank control group (without polysaccharides), LPS and polysaccharide samples (50, 100, 200, 400, and 800 μg/ml). After incubation for 24 h, the proliferation activity was determined using the CCK-8 method (24). Each concentration was repeated six times in the well. RAW264.7 cells (5 × 10^4^ cells/ml) were seeded into a 96-well flat-bottom plate and cultured for 12 h. Then, samples (100 μg/ml: the optimal concentration screened from proliferation activity) were added, followed by another 24 h incubation. After that, the phagocytosis activity was determined by the neutral red staining method (25). Each concentration was repeated six times in the well.

#### Quantitative analysis of NO and cytokines

RAW264.7 cells (1 × 10^5^ cells/ml) were seeded into a 24-well flat-bottom plate and cultured for 12 h. Then, control, LPS, and polysaccharide samples (100 μg/ml) were added to cells. After incubation for another 24 h, the cultured supernatants were collected (26). The quantifications of cytokines TNF-α, IL-6, and IL-10 were measured using commercial ELISA kits. The total NO content were measured using the Nitric Oxide Assay Kit according to the manufacturer’s instructions (Nanjing Jiancheng Institute of Biotechnology, China).

#### RT-qPCR analysis

RAW264.7 cells (1 × 10^6^ cells/ml) were seeded into a six-well flat-bottom plate and cultured for 12 h. Then, control (blank), LPS, and polysaccharide samples (100 μg/ml) were added to cells. After incubation for another 24 h, the total RNA of the cultured cells was isolated using kit (OMEGA, USA), and then cDNA was immediately synthesized using a reverse transcription kit (Thermo, USA). The specific primers (Supplementary Table 1) for RT-qPCR were designed to amplify a portion (nucleotides about 150-bp) of the 3′ end of the target genes to analyze the mRNA-expression levels of IL-6, IL-10, and TNF-α in RAW264.7 cells (23). The amplification conditions were PCR initial activation step (95°C for 30 s), and 40 cycles of denaturation (95°C for 5 s), annealing (60°C for 30 s), and extension (72°C for 60 s) using the Stratagene Mx3000P thermocycler (Applied Technologies, USA). Expression of gene was measured in triplicate, and was analyzed via 2^–△△CT^ method (8).

### Statistical analysis

The results were presented as mean ± SD (standard deviation). In addition, Duncan’s test and one-way analysis of variance (ANOVA) were used for multiple comparisons by the SPSS 20.0 software package.

## Results and discussion

### Structural characterization

#### Degree of de-acetylation (DD) by the hydrolysis method

The contents of neutral sugar and protein in DOP were determined to be 89.21 wt% and 3.12 wt% in dry base, respectively, indicating DOP has a good purity. In addition, DOP contained 6.84 wt% acetyl groups, which has been reported to be vital for the good water solubility of glucomannan (27). In our study, Na_2_CO_3_ treatment significantly decreased the DA of DOP. The DD value was increased from 39.33 to 86.84% when the Na_2_CO_3_ treatment duration was elevated from 3 to 25 min (Table 1). The addition of alkali plays an efficient role in facilitating the de-acetylation of the molecular chain. Similarly, Li et al. (28) reported that alkali concentration and treating time were both positively related to the DD of the konjac glucomannan. In contrast to Na_2_CO_3_ treatment, ultrasonic treatment only slightly decreased the DA of DOP. The DD value followed the sequence of US-60 (12.13%) < US-200 (26.61%) < US-720 (39.69%), suggesting that ultrasound favored chain degradation over the cleavage of the acetyl groups.

**TABLE 1:** The structural characterization of native and modified DOPs.

	DOP	US-60	US-200	US-720	DA-3	DA-5	DA-25
DA (%)	6.84 ± 0.06^a^	6.01 ± 0.01^b^	5.02 ± 0.03^c^	4.12 ± 0.11^d^	4.15 ± 0.21^d^	2.54 ± 0.06^e^	0.90 ± 0.14^f^
DD (%)	0^a^	12.13 ± 0.21^b^	26.61 ± 0.41^c^	39.69 ± 1.55^d^	39.33 ± 3.10^d^	62.87 ± 0.83^e^	86.84 ± 2.07^f^
Solubility (%)	57.5 ± 2.2^a^	63.6 ± 1.8^b^	68.9 ± 2.7^c^	78.2 ± 2.1^d^	28.7 ± 1.1^e^	21.1 ± 1.9^f^	3.2 ± 0.6^g^
**Molecular parameters**							
Mn (kDa)	47.3	43.7	27.7	23.7	56.5	58.5	19.9
Mp (kDa)	60.2	48.6	32.4	28.7	67.5	70.3	16.5
Mw (kDa)	85.4	58.0	34.2	30.2	95.9	106.7	56.2
PDI = (Mw/Mn)	1.8	1.3	1.2	1.3	1.7	1.8	2.8
**Linkage patterns (mol%)**							
→4)-Man*p*-(1→	91.4	91.6	91.5	91.4	92.7	91.6	92.8
→4)-Glc*p*-(1→	8.6	8.4	8.5	8.6	7.3	8.4	6.2

Data are mean_±_ SD of three replicates.

Data in the same row with different letters indicated significant differences at *p* < 0.05.

#### Mw analysis

**FIGURE 1:**
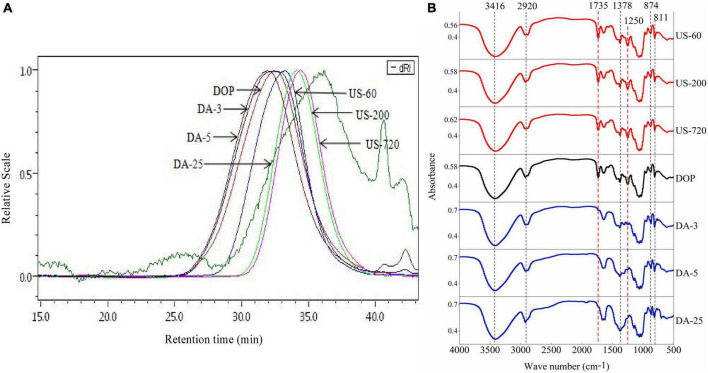
HPSEC elution profiles **(A)** and Infrared spectra **(B)** of the native and modified DOPs.

The molecular weights of DOP before and after modification were obtained from multi-detector HPSEC analysis. As shown in Table 1 and Figure 1A, except for DA-25, all the other modified DOPs showed a relatively low Mw distribution after treatment as indicated by the low polydispersity index (PDI = Mw/Mn) value. Samples derived from the ultrasonic treatment decreased in the Mw order of DOP (85.4 kDa) > US-60 (58.0 kDa) > US-200 (34.2 kDa) > US-720 (30.2 kDa). The Mw of DOP reduced by 32.1, 60.0, and 64.6% after treatment for 60, 200, and 720 min, respectively. The results indicated that ultrasonic treatment could effectively degrade DOP, and higher treating time led to the lower Mw until a plateau was reached. Similar results have also been reported for schizophyllan (29), dextran (30), apple pectin (31), and polysaccharides from the seeds of *Plantago asiatica* L. (32). The influence of ultrasound on the degradation of the polysaccharides ascribes to the cavitation action, which involves two mechanisms: mechanical degradation of the polysaccharide from collapsed cavitation bubble and chemical degradation because of the chemical reaction between the polysaccharide and high-energy molecules, e.g., the hydroxyl radicals produced during cavitation (33). In our study, the Mw of Na_2_CO_3_ treated samples increased slightly compared to the natural DOP, which was likely caused by the conformational change induced by de-acetylation treatment as well as the formation of aggregation. This was consistent with the Mw data reported by Salah et al. (34).

#### Glycosidic linkage patterns

The linkage patterns of DOP before and after modification, derived from the methylation analysis, are compared in Table 1. According to the methylation analysis result of DOP, there were two main glycosidic linkage patterns, (1→4)-D-Man*p* and (1→4)-D-Glc*p* with a molar ratio of nearly 10.6:1.0. Ultrasonic and mild base treatments did not change the overall monosaccharide composition and linkage patterns. This finding indicated that ultrasonic and de-acetylation treatments had no major impact on the monosaccharide composition and linkage patterns of polysaccharides, which confirmed the previous findings by Dou et al. (35) and Wang et al. (36).

#### FT-IR analysis

The structural changes of DOP before and after modification are compared using FT-IR (Figure 1B). The intensified and broad absorbance bands at around 3,416, 2,920, 1,378 cm^–1^ were attributed to O–H hydroxyl stretching vibration, C–H stretching vibration of the methyl group, the symmetric C–H bending vibration of the methyl group, respectively (6). The band at approximately 1,735 cm^–1^ was assigned to the valence vibration of C=O (37), and the strong peak at 1,250 cm^–1^ indicates the presence of C–O vibration of O-acetyl groups, confirming the presence of acetyl groups (4). The two specific bands at 811 and 874 cm^–1^ confirmed the existence of mannan in DOP, as expected (38). For the modified samples, these characteristic peaks were preserved after ultrasonic treatment, suggesting ultrasound treatment had no clear alteration in the functional group of DOP. However, Na_2_CO_3_ treatment weakened the two absorption bands at 1,735 and 1,250 cm^–1^. The disappearance of the signal peak at 1,735 cm^–1^ in the de-acetylated polysaccharide samples (DA-3, DA-5, DA-25) indicated the mild base treatment disrupted the C=O double bond of the acetyl group. Meanwhile, the relative peak intensity of 1,250 cm^–1^ (C–O vibration of O-acetyl groups) signal decreased with increasing DD, double confirming this deduction.

It can be herein concluded ultrasonic treatment significantly decreased the Mw of DOP while mildly affecting DA. The DA of DOP reduced significantly after mild base treatment for 3 (DA: 4.15%, DD: 39.33%), 5 (DA: 2.54%, DD: 62.87%), and 25 min (DA: 0.90%, DD: 86.84%). Both treatments did not affect the overall monosaccharide composition and glycosidic linkages of DOP.

### Water solubility

Using the centrifugation method, the water solubility of native DOP at room temperature was 57.5% (Table 1), which respectively increased to 63.6, 68.9, and 78.2% under 60, 200, and 720 min of ultrasonic treatment. This result was in accordance with our previous study on arabinoxylan (12), which implied that the decrease in molecular size due to the breakage of glycosidic bonds and chain scission during ultrasound treatment improved the water solubility of polysaccharide molecules (33).

In contrast, increasing Na_2_CO_3_ treating times in de-acetylation reaction conferred a negative effect on the water solubility. As shown in Table 1, upon Na_2_CO_3_ concentration of 0.2 M and hydrolysis time of 25 min, the insoluble fractions significantly increased, resulting in the solubility of the DA-25 being only 3.2%. The decrease in solubility could be due to the loss of acetyl groups, which decreased the intermolecular steric hindrance, thereby increasing the intermolecular interaction through hydrogen bonding and decreasing the solubility (27). Our results are well matched with the report from Chokboribal et al. (39) who found that de-acetylation of acemannan from *Aloe vera* decreased water solubility. They articulated that de-acetylation reduced the steric hindrance of molecular chain and increased crystalline structure which had lower solubility.

### Conformational characterization

The conformation of polysaccharide molecules dictates their three-dimensional shape in solid-state or in solutions, such as spherical, random coil, double-helix, triple-helix, worm-like, rod-like. In this study, the chain conformation of native and modified DOPs was studied to understand the influences of structural modifications on their conformational properties. The parameters Rhz (z-average hydrodynamic radius), Rgz (z-average radius of gyration), and [η]_*w*_ (weight-average intrinsic viscosity) obtained from multi-detector HPSEC are presented in Table 2. The values of Rhz, Rgz and [η]_*w*_ for DOP were determined to be 21.4 nm, 32.4 nm, and 264.2 mL/g, respectively. However, these molecular parameters decreased after ultrasonic treatment, implying that ultrasonication disrupted the polymer aggregates and cleaved the polymer chains in solution (40). For the mild base treatment, their parameters Rhz, Rgz, and [η]_*w*_ were increased, which were likely attributed to the removal of the acetyl group.

**TABLE 2:** Conformational characterizations of native and modified DOPs.

	DOP	US-60	US-200	US-720	DA-3	DA-5	DA-25
_*w*_(ml/g)	264.2	199.5	127.5	115.6	287.8	299.7	101.0
Rhz (nm)	21.4	13.8	10	9.2	22.1	28	13.2
Rgz (nm)	32.4	24	17.7	16.8	33.0	32.8	27.9
ρ(= Rg_*z*_/Rh_*z*_)	1.51	1.74	1.77	1.83	1.49	1.17	2.11
**Mark-Houwink-Sakurada equation: [η] = K_η_ M^α^**							
α	0.64	0.86	0.86	0.90	0.80; 0.35	0.80; 0.26	–
logKη	–0.70	–1.77	–1.79	–1.97	–1.50; 0.93	–1.44; 1.38	–
**Conformational power-law equation: Rg = K_*g*_M*^v^***							
*v*	0.57	0.63	0.65	0.69	0.52; –0.10	0.51; –0.12	–
logKg	–1.45	–1.70	–1.75	–1.92	–1.16; 2.27	–1.10; 2.32	–

The characteristic parameter ρ (Rg/Rh) has been previously used to reflect the conformational properties of polysaccharides (Table 2). The different ρ values reflect various molecular conformations, ρ ≥ 2.00 for rigid chains (cylinders), 1.50–1.80 for random coils in a good solvent while 1.30 in a θ solvent, 1.00–1.11 for loosely hyper-branched chains and 0.78 for compact spheres (41). Our study calculated the characteristic parameter ρ of DOP as 1.514, indicating that DOP was a random coil conformation. After ultrasonic treatment, the ρ value was in the range of 1.5–1.8, which can be assigned to flexible coil conformations. In addition, an increasing chain rigidity was noticed for ultrasonication treated samples with increasing duration (DOP < US-60 < US-200 < US-720), showing the lower the Mw of fractions, the more rigid the molecular chain for DOP. For mild base treatment (de-acetylation treatment), the ρ value gradually decreased with decreasing DA, implying that the chain conformation turned more compact from DA-3 to DA-5.

**FIGURE 2:**
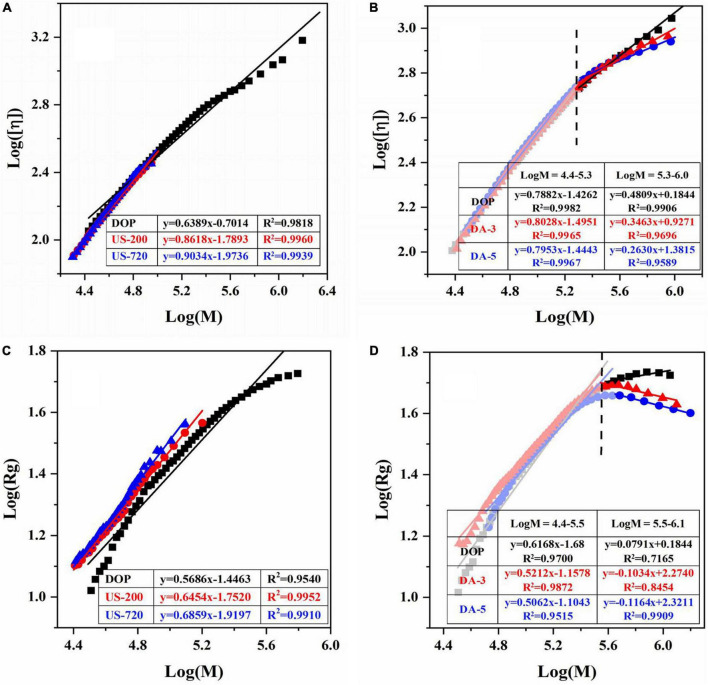
Logarithmic plot of the molecular weight vs intrinsic viscosity, radius of gyration of native and modified DOPs in aqueous solution. The Mark-Houwink plots of ultrasound **(A)** and de-acetylated **(B)** samples. The Rg power-law plots of ultrasonic **(C)** and de-acetylated **(D)** samples.

Furthermore, the relationships of Mw with cumulative weight fraction, [η] (Supplementary Figure 1), and Rg (Supplementary Figure 2) were established. The double logarithmic plot of the [η]-Mw and Rg-Mw have been well described using the Mark-Houwink ([η] = *k*_η_*M*^α^), conformational power-law equation (*Rg* = *k*_*g*_*M^v^*), respectively (40, 42). For the native DOP, a good linear regression (R^2^ = 0.9818) was found between [η] and Mw in the log Mw range of 4.4–6.3, the slope α was obtained as 0.6389 (Figure 2A), indicating that DOP was flexible random coil conformation. As expected, the chain conformation became gradually rigid while increasing ultrasound time and decreasing molecular weight or molecular size (Figure 2B). In terms of the mild base (de-acetylation) treatment, it is worth noting the curves in Figure 2B were not completely linear, i.e., a decreased slope was observed in DA-3 or DA-5 with increasing Mw. Hence, two linear regressions were used to fit the curve. The slope (α) had no clear change in the log Mw range of 4.4–5.3, suggesting the destruction of acetyl groups may not mainly occur on low molecular weight polysaccharides. However, in the high logMw range of 5.3–6.0, with the decrease of DA value, α value gradually decreases, indicating that DA-3 (0.3463) / DA-5 (0.2630) exhibited more compact chain conformation in the high Mw range, likely as the result of the reduction in steric effects from the acetyl groups. Among the α values of these modified samples, DA-25 did not show a good linear regression due to the poor solubility.

The value of *v* for DOP was 0.5686 in the logMw range of 4.4–5.8, confirming that the native DOP had a random coil conformation in an aqueous solution. After ultrasound treatment (Figure 2C), the chain conformations for US-60 (0.6306), US-200 (0.6454), US-720 (0.6859) became more rigid with increased treating time, in accordance with the results of Mark-Houwink equation. After de-acetylation treatment (Figure 2D), the changes of *v* values showed a similar trend to that of α, which double confirmed the previous deduction.

Based on the above conformational analysis, native DOP demonstrated a typical flexible random coil chain conformation in solution. The coil conformation became rigid when decreasing the Mw, while with the decrease of DA value, the molecular chain turned more compact coil conformation, especially for high Mw fractions.

### Immunomodulatory activities

#### The proliferation and phagocytosis activities

Macrophages, the primary effector cells of innate immune, play a critical role in immediate response against pathogens. In this study, RAW264.7 macrophages were applied to investigate the direct effect of molecular modification on immunomodulatory activities.

**FIGURE 3:**
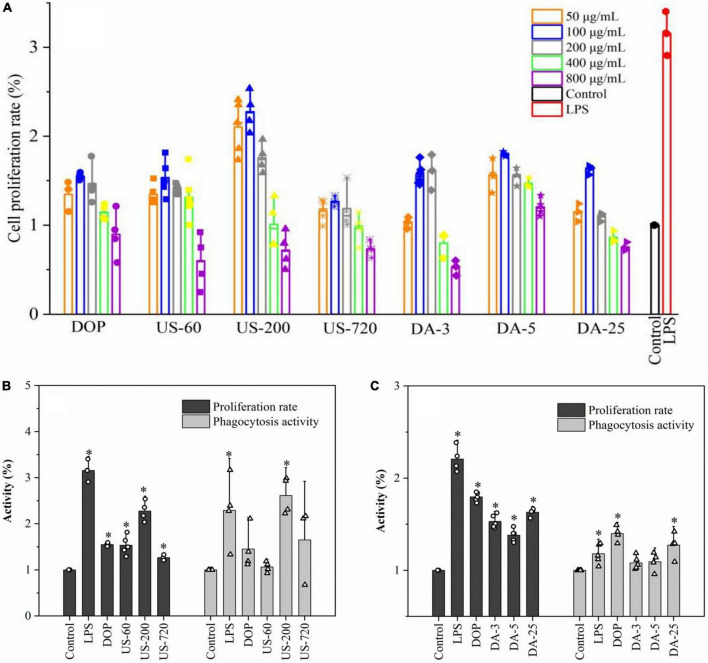
Polysaccharide induced proliferation and phagocytosis of macrophages. **(A)** Determination of optimal concentration (50, 100, 200, 400, 800 μg/ml). The proliferation and phagocytosis activities of ultrasonic **(B)** and de-acetylated **(C)** samples (100 μg/ml). All data are representative of at least three independent experiments. *p < 0.05 vs. control.

Cell viability was first examined to ensure that the sample dosages used were not toxic to cells, and the results are presented in Figure 3A. Compared to the control, samples showed positive effects on proliferation activity in 50–400 μg/ml, but the cell viability was suppressed at 800 μg/ml. In addition, the cell viability of polysaccharide samples reached the highest at 100 μg/ml compared to other concentrations. Therefore, 100 μg/ml was selected as the optimum dosage for other immunological tests.

The cell proliferation rate and phagocytosis activities of native and modified DOPs are presented in Figures 3B,C. Under polysaccharide stimulation, proliferation and phagocytosis activities were significantly increased in RAW264.7 macrophages. US-200 demonstrated significantly higher proliferation and phagocytosis activities than DOP, US-60, and US-720, indicating DOP with a specific molecular size (weight) range can exert better activity. However, the immunomodulatory effect of glucomannan was compromised when the Mw was too low. In terms of the mild base treated samples (de-acetylation), the proliferation and phagocytosis activities were significantly decreased compared with the native DOP. It was concluded that DOP with a DA of 6.84% exerted better activity, indicating the acetyl group is one of the key factors in the cell activities. Simões et al. demonstrated that acemannan with a higher degree of acetylation had better biological activity, and this activity decreased with decrease degree of acetylation (43). Moreover, Chokboribal et al. (39) indicated that the bioactivity of acemannan was reduced after de-acetylation. These data well matched with our finding that glucomannan with a higher degree of acetylation had increased biological activity.

#### Effects on cytokine production

**FIGURE 4:**
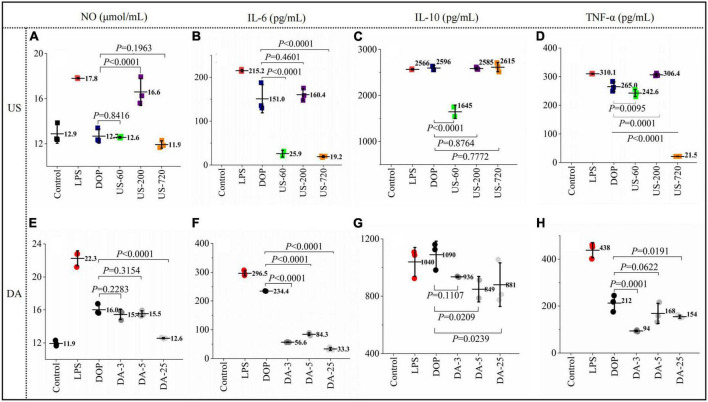
Polysaccharides induced production of immune factors NO **(A,E)**, IL-6 **(B,F)**, IL-10 **(C,G)**, TNF-α **(D,H)** productions (24 h) by macrophages RAW264.7 exposed to ultrasonic and de-acetylated samples (100 μg/ml) were assessed by ELISA. All data are representative of at least triplicate culture.

Macrophage activation by immunomodulators induces several signaling pathways to produce various immune factors, triggering related immune responses. The effects of native and modified DOPs on macrophage NO, TNF-α, IL-6, and IL-10 production were analyzed in this study. As shown in Figure 4, compared to the blank control, incubating RAW264.7 cells with LPS and various DOP fractions significantly increased the secretions of TNF-α, IL-6, IL-10, and NO.

For the ultrasonication treated DOP, US-200 showed the highest NO concentration (16.59 μmol/ml), which was increased by 30% compared to DOP (Figure 4A). Similarly, US-200 showed the highest positive effects on IL-6 (Figure 4B), IL-10 (Figure 4C), and TNF-α (Figure 4D) production, possibly attributed to the appropriate molecular size and DA value. ELISA analysis showed that the cellular release of NO (Figure 4E), IL-6 (Figure 4F), IL-10 (Figure 4G), and TNF-α (Figure 4H) were reduced after mild base treatment, indicating the loss of acetyl group compromised the related immune effects. Therefore, the presence of acetyl groups in DOP could directly affect and regulate their bioactive properties. Similar results have also been reported for acemannan from *Aloe vera* (34).

#### Effects on mRNA expression

**FIGURE 5:**
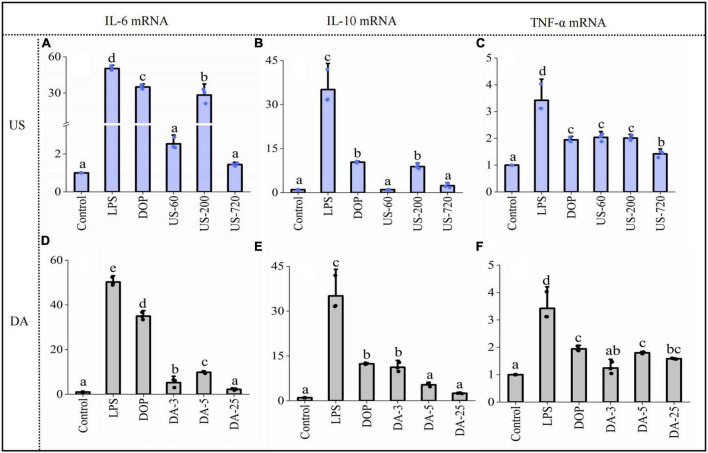
Polysaccharides induced gene expression of cytokine IL-6 **(A,D)**, IL-10 **(B,E)**, TNF-α **(C,F)** productions (24 h) by macrophages RAW264.7 exposed to ultrasonic and de-acetylated samples (100 μg/ml) were assessed by RT-qPCR. Data are expressed as means plus SD of triplicate culture (different letters indicated significant differences at *p* < 0.05).

Stimulation of immunomodulators leads to the initiation of intracellular signaling pathways and eventually induces transcriptional activation and expression of cytokines. The expression of cytokines, including TNF-α, IL-10, and IL-6 is essential for host survival from infection. In addition, they are recognized as important host defense molecules that affect tumor cells. To determine whether the increases in IL-10, and IL-6, and TNF-α secretion are attributable to the facilitated gene expression of IL-10, and IL-6, and TNF-α. Cells were treated with 100 μg/ml samples for 24 h, then cytokine mRNA levels were measured by RT-qPCR. The results showed that cytokines mRNAs were barely detectable in unstimulated RAW cells. After co-cultured with native and modified DOPs, the mRNA levels for all these immune factors increased significantly. Moreover, the expression of IL-6 (Figure 5A), IL-10 (Figure 5B), and TNF-α (Figure 5C) treated by US-200 was highest among all samples. By contrast, the de-acetylated fractions (DA-3, DA-5, and DA-25) significantly reduced the mRNA expression of cytokines, confirming the O-acetylation level is one of the critical determinants of immunomodulatory (Figures 5D–F).

It was well known that TNF-α and IL-6 are pro-inflammatory cytokines and IL-10 was an anti-inflammatory cytokine. Therefore, the present data indicated that DOP exhibited both pro-inflammatory and anti-inflammatory activity. This dual-direction regulation was reported for other polysaccharides (44, 45), which indicated that the immunomodulatory activity of polysaccharides was a complicated process and needed to be further studied.

**FIGURE 6:**
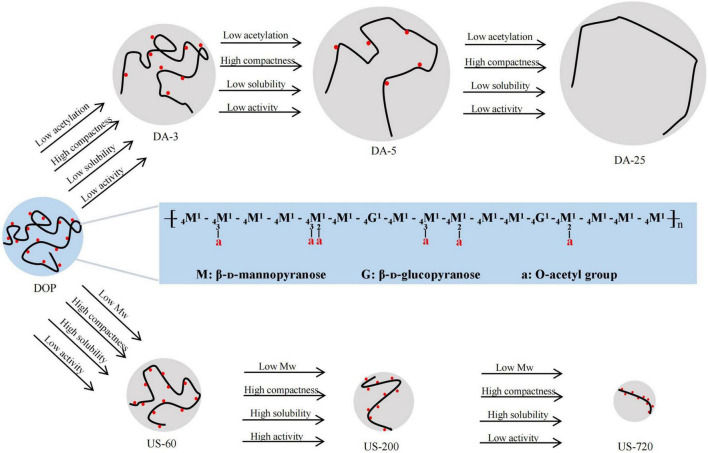
Schematic diagram of structural-conformational-immunomodulatory relationships of DOP The major structure of DOP adapted from Xing et al. (7).

We confirmed the acetyl group was one of the critical determinants of immunomodulatory to modulate the immune system. As demonstrated by Scully et al. (46), O-acetylation is necessary to induce effective opsonophagocytic killing responses. Similarly, Kumar et al. (47) reported that the activation effect of native or over-acetylated or de-acetylated acemannan on macrophages, the results showed that removal of O-acetyl groups resulted in a lower immunomodulatory, while the over-acetylated polysaccharide has stronger effects on immunomodulation. It has also been demonstrated that ultrasound decreased the Mw and improved the immunomodulatory activity of DOP. Many studies also proved that ultrasonic degradation is an effective way to improve various bioactivities of natural polysaccharides (32, 33, 35). However, the immunomodulatory effect of glucomannan was compromised when the Mw was too low, suggesting the immune cells can only be activated if the Mw or molecular size of DOP was large enough. It is worth pointing out that low degree of acetylation and low Mw can confer good solubility property and induce compact coil conformation of glucomannan. Some specific physicochemical parameters, such as degree of acetylation of glucomannan, proper ranges of Mw, water solubility and molecular conformation, all shed lights on the immunomodulatory effects (Figure 6). The effects may be correlated with a receptor-ligand binding in macrophages, given that glucomannan has been reported to trigger the activation of immune cell through the interactions with TLR4 receptor (8), TLR2 receptor (48), TLR22 receptor (49), or mannose receptor (50, 51).

## Conclusion

Based on our study results, ultrasound is conducive to chain degradation rather than acetyl cracking, and the acetylation level of sonicated samples only changed slightly. Na_2_CO_3_ treatment is conducive to de-acetylation rather than chain degradation, the Mw of Na_2_CO_3_ treated samples changed slightly. Thus, samples with different molecular weights and degrees of acetylation were obtained by ultrasound and alkali treatments to establish the structural-immunomodulatory relationships. As a result, DOP with a higher degree of acetylation had increased biological activity, and this activity reduced with decreasing DA, indicating the O-acetylation level was one of the critical determinants of immunomodulatory to modulate the immune system. In addition, slightly reduced the Mw of DOP (US-200, Mw of 34.2 kDa) significantly increased immune-regulation effects of DOP. However, the results also showed that the immunomodulatory effect of glucomannan was compromised when the Mw was too low. Acetyls and low Mw confer the solubility property and compact coil conformation of glucomannan. The specific physicochemical parameters, such as degree of acetylation of glucomannan, proper ranges of Mw, water solubility and molecular conformation, all make contribution to their immunomodulatory effects.

## Data availability statement

The original contributions presented in this study are included in the article/Supplementary material, further inquiries can be directed to the corresponding authors.

## Author contributions

XG and QG conceived and designed the research, and wrote the manuscript. XG and MY conducted the experiments. CW, SN, and SC supervised and revised the manuscript. All authors contributed to the article and approved the submitted version.
