# Supplementary material for: Acetyl-glucomannan from Dendrobium officinale: Structural modification and immunomodulatory activities
Source: Front Nutr. 2022 Sep 29;9:1016961. doi: 10.3389/fnut.2022.1016961 (PMC9558108; doi:10.3389/fnut.2022.1016961)
Supplement: Supplementary file 1 [file Table_1.DOC]

**Supporting Information**


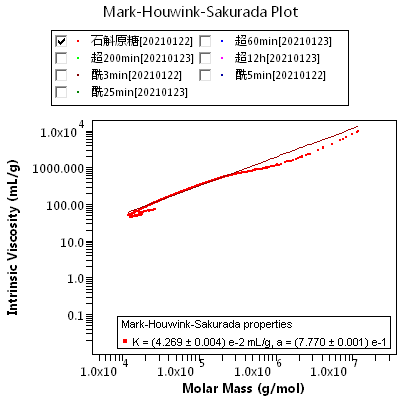

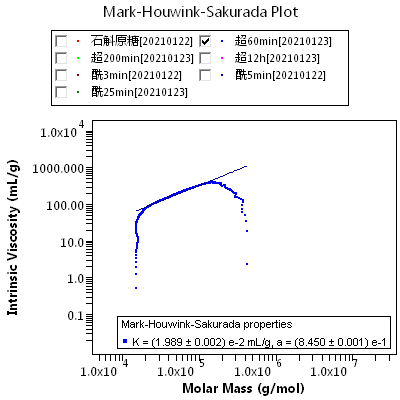


DOP

US-60


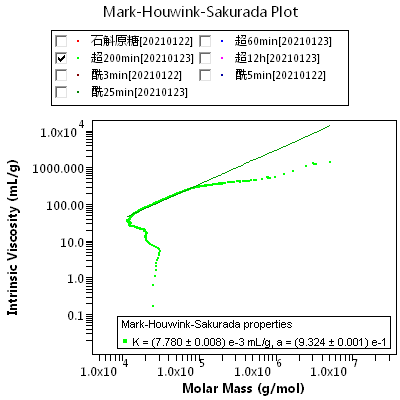

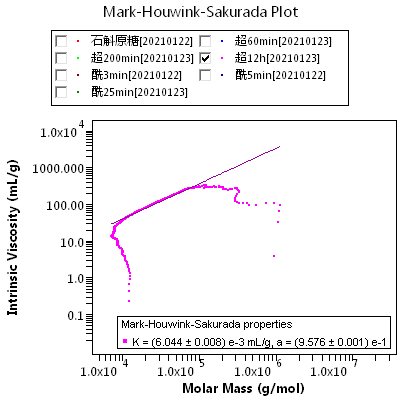

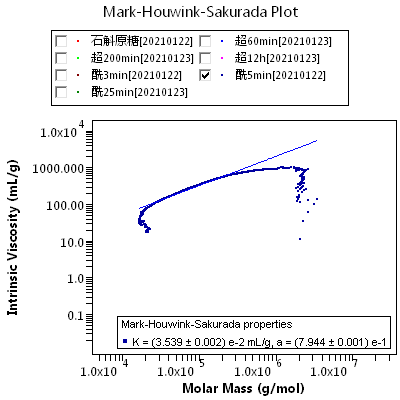

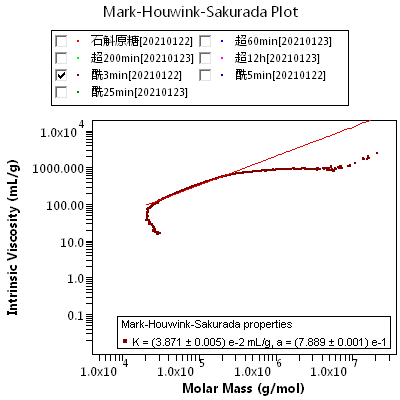


DA-3

US-200

US-720

DA-5


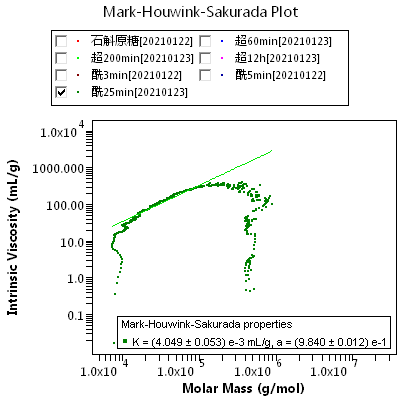


DA-25

**Fig. S1** Plots of [η] vs Mw of the native and modified DOPs.


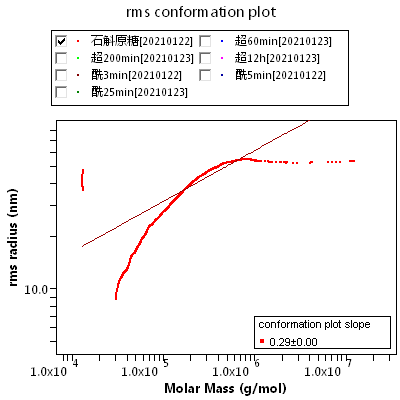

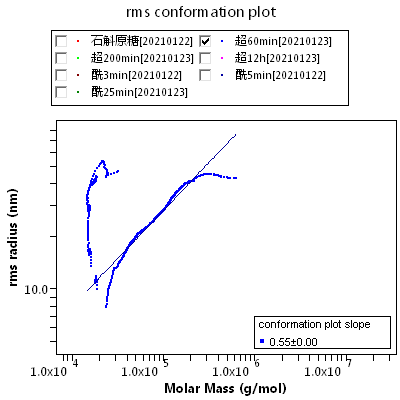


DOP

US-60


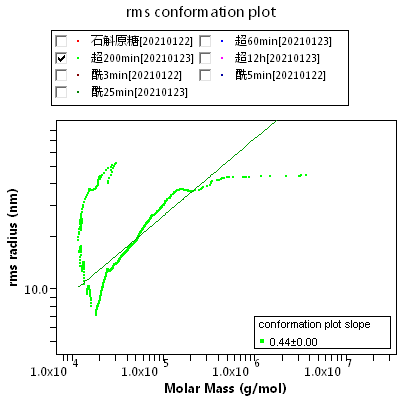

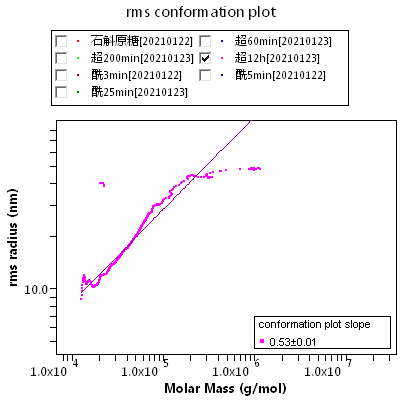


US-200

US-720


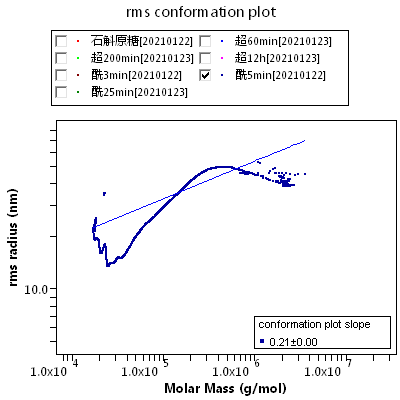

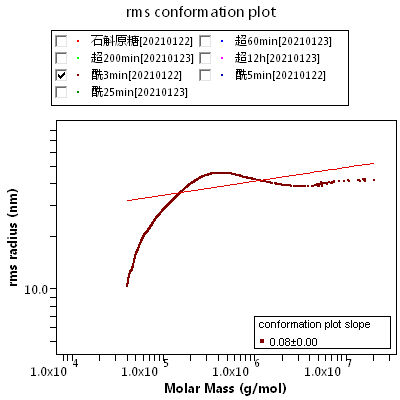


DA-3

DA-5


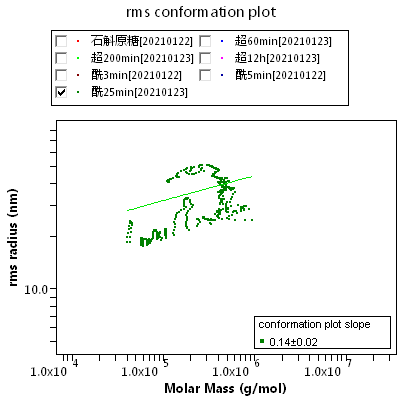


DA-25

**Fig. S2** Plots of Rg vs Mw of the native and modified DOPs.

Table S1. Primer sequences of RT-qPCR analysis

| Primer | Sequence (5'to3') ^a^ |
| --- | --- |
| TNF-α | F: AAGTTCCCAAATGGCCTCCC  R: TTGCTACGACGTGGGCTAC |
| IL-6 | F: ATCCAGTTGCCTTCTTGGGA  R: GGTCTGTTGGGAGTGGTATCC |
| IL-10 | F: GGTTGCCAAGCCTTATCGGA  R: TCAGCTTCTCACCCAGGGAA |
| β-actin | F: CGTAAAGACCTCTATGCCAACA |
|  | R: ACAGTCCGCCTAGAAGCAC |

^a^ The forward (F) and reverse (R) primer pairs.
